# Goats worm burden variability also results from non-homogeneous larval intake

**DOI:** 10.1038/s41598-018-34338-2

**Published:** 2018-10-30

**Authors:** Mathieu Bonneau, Jean-Christophe Bambou, Nathalie Mandonnet, Rémy Arquet, Maurice Mahieu

**Affiliations:** 1 INRA - URZ, UR143, Petit-Bourg, 97170 Guadeloupe, French West Indies France; 2 INRA - UE PTEA, UE1294, Petit-Bourg, 97170 Guadeloupe, French West Indies France

## Abstract

For small ruminants, Gastrointestinal Nematodes (GINs) are responsible for severe economic losses and they are also an animal welfare problem. GIN use their host to reproduce and disperse eggs on the pasture, from where they can re-infect another animal. The high density of hosts on the pasture and the extreme tolerance of GIN to environmental constraints make GIN eradication almost impossible. In addition, significant resistance to anthelmintic treatment requires sustainable and integrated management to maintain the health and financial well-being of livestock farming. In this context, models of the complex interactions between host, GIN and environment can help us to design long term optimal management strategies. To build such models, quantitative information is needed but are generally very challenging to collect. In this article, we focus on the number of ingested larvae per animal, which we propose to characterise by using a simulation framework based on the estimation of the spatial distribution of the host over time. Our framework allows us to show that worm burden individual variation is not only explained by the host’s genetics, as is often the case, but is also a result of the grazing spatial process.

## Introduction

Gastrointestinal Nematodes (GINs) are a major threat to the health and welfare of small ruminants and they can be responsible for severe economic losses^1^. For any grazing animals, infection by GINs is an almost inevitable event, but the severity during lifetime and the animal’s reaction varies. While the causes explaining this variability are mostly known–they can be intrinsic (genetics, age, sex, life history or physiological stage) and extrinsic (climate, nutrition or farmer’s practices)–quantifying their effects on the success of an infection event and its consequences is very challenging.

The life cycle of GINs is a succession of free living stages (FL) and parasitic stages (P). During (FL), GINs are found on the pasture, where they were dropped in the feces of infected animals. GINs start as eggs, and they eventually hatch and evolve into infective L3 that can survive for a relatively long period of time depending on meteorological factors, such as temperature and humidity^2–4^. Parasitic stages (P) start when the host ingests infective L3, which eventually establish inside the host and develop into reproductive adult GINs. Reproduction of the species is only possible inside the host, where new eggs are laid with the feces and dropped on the pasture. Control and research efforts mostly focus on the parasitic stages, where it is possible to kill the GINs, either using chemicals or by improving host’s resistance. In this article, we are interested in the meeting point between (FL) and (P): the ingestion of L3 and more precisely to study the distribution of the ingested L3 among the individuals of a same flock.

The timing and quantity of ingested L3 are important because they can drive the future resistance status of an individual^5^ and affect the population dynamic of parasites, as in the *periparturient rise of fecal eggs count*^6^. In addition, L3 are known to have a clumped distribution on the pasture^7^, which could be responsible for the aggregated distribution of parasites among the flock^8^ and helps to spread resistant parasites to anthelmintic treatments^9,10^. Although quantitative data can be useful to improve control, they are quite hard to collect. Indeed, estimating the spatial distribution of L3 on the pasture is highly time consuming and it is technically difficult to observe the quantity of ingested L3 during natural grazing^11^, to such an extent that a modeling approach is needed. The timing and quantity of ingested L3 are the result of two correlated spatial processes: (L) the spatial distribution of L3, resulting itself from (D) the spatial distribution of animals over time. Foraging behavior^12^ can explain part of (D) and has been used to define and parameterise models describing the displacement of small ruminants over time^13–15^. Even if it is possible to use these models to simulate animals displacement and feces deposit to study parasite transmission^16^, modeling an animal’s decision is inevitably a difficult task, particularly because not only feeding influence displacement over the day, but also environmental factors and social interactions, not accounted for in these models. In our study, instead of relying on foraging behavior theory, we proposed relying directly on (D), which is the flock’s spatial distribution over time.

We used drone imagery to record the positions of goats, who were managed under rotational grazing. We recorded the position of two flocks (i.e., two replicas) during their first week on their respective pasture. Making the hypothesis that the likelihood of dropping feces clump on a given position increases with the time that the animal spends on that position, we were able to simulate spatial distribution of L3 when the flock re-entered the pasture one month later. The individual positions were also recorded during this second week, which we used this time to estimate the ingestion risk, making the hypothesis that ingestion risk increases with the time spend on a position × the number of L3 present on the herbage. We hypothesised that ingestion risk and number of ingested L3 are proportional and that information on the risk distribution can also be used to characterise the distribution of the number of ingested L3. Our framework is entirely stochastic.

We first proposed to characterise the risk distribution at the individual and flock scales, starting by analyzing the effect of specificity on the individual risk distribution. The specificity parameter *a* ∈ [0.001; 0.1] describes how a goat explore the pasture, spending many (few) times on few (many) quadrats when *a* is high (low). Second, we characterised the spatial distribution of the feces. We finally used our framework to discuss the effect of the number of individuals on a pasture.

## Results

### Simulation model

**Figure 1:**
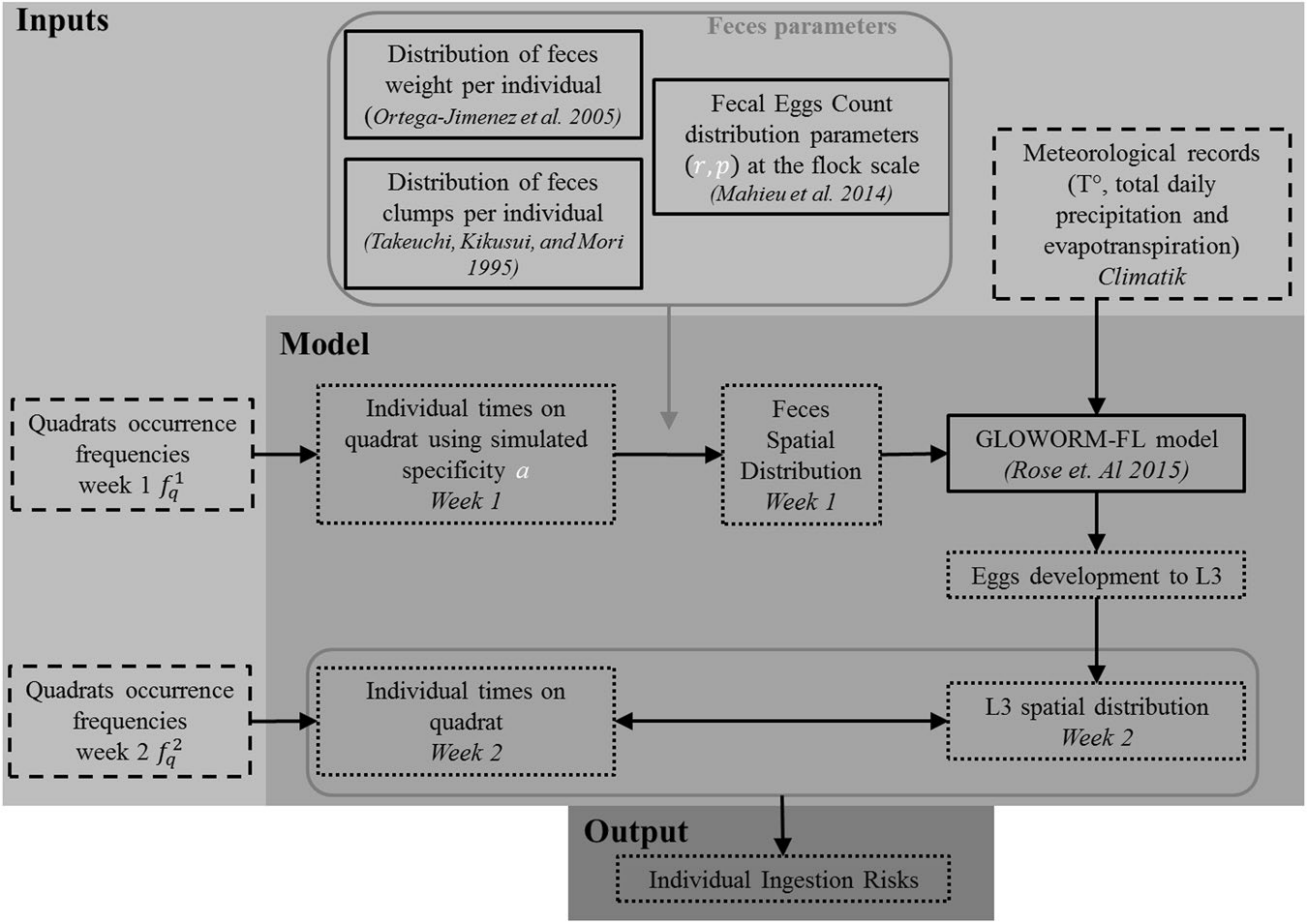
Schematic representation of the model inputs and output. Boxes with solid lines indicate data or model from the literature. Boxes with dashed lines indicate data recorded during the experimentations. Boxes with dotted lines indicate data simulated by the model.

The simulation model is summarised in Fig. 1.

The model’s inputs are the feces parameters, elicited from the literature, the quadrats occurrence frequencies at the flock scale during the first and second grazing week (two replicas), as well as meteorological data, which were both recorded during a dedicated experiment. The model then simulated the individual time spent on each quadrat during the first and second grazing week, the feces deposit during first week and L3 spatial distribution at the beginning of the second grazing week. The model is finally used to simulate an individual L3 ingestion risk.

### Risk distribution

Unless otherwise specified, our conclusions are the same for the first and second flock. In the following, initial Fecal Eggs Count (FEC) distribution refers to the distribution, or more precisely parameters (*r*, *p*), that we used to simulate the FEC of each animal at the beginning of a simulation (i.e., when goats enter the pasture during week 1). We recall that initial mean FEC is equal to $\frac{r\mathrm{(1}-p)}{p}$.

### Impact of specificity

In the following, a sample is the 1,000 simulated risk values of the first goat *g* = 1, for a given specificity and initial FEC distribution.

**Figure 2:**
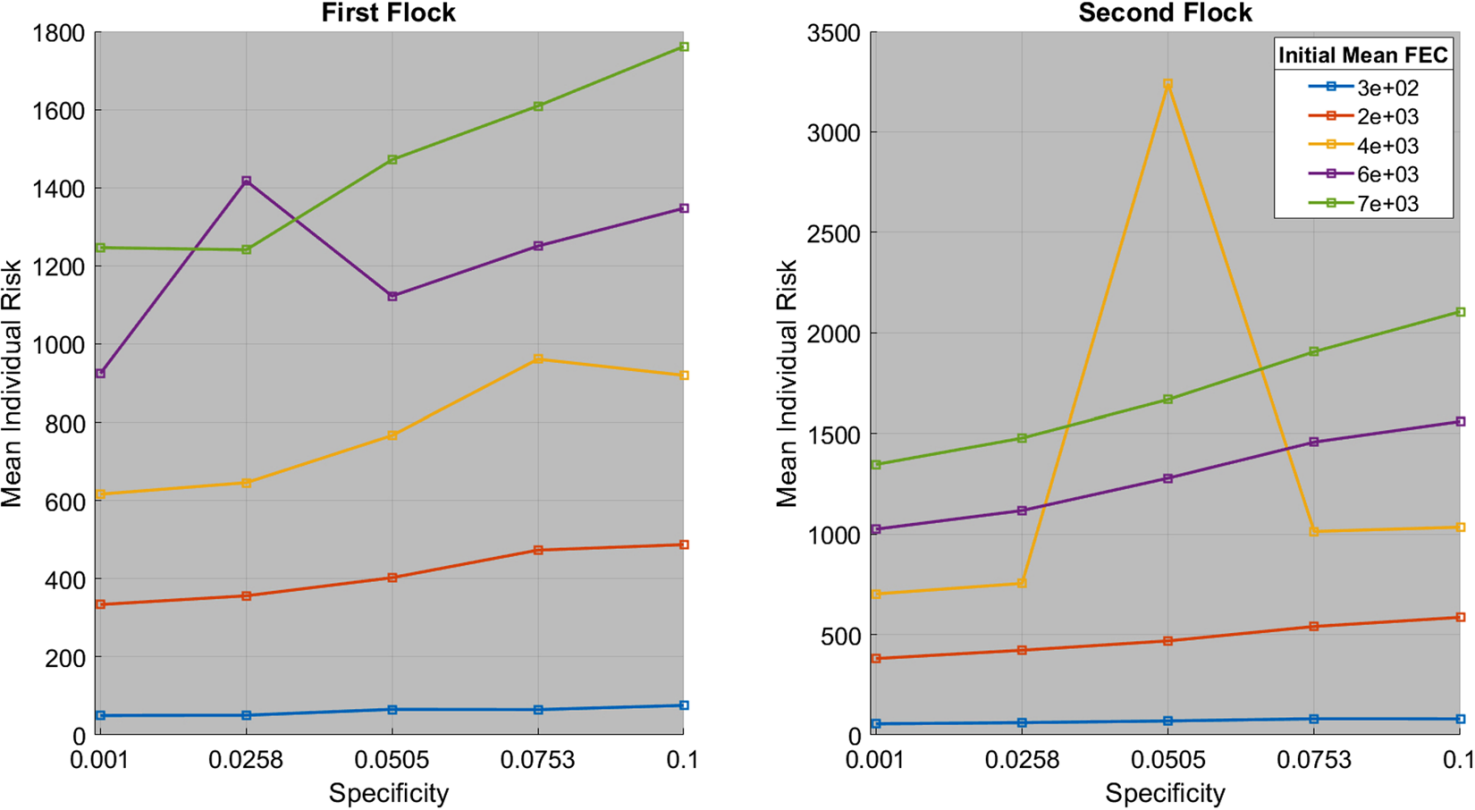
Mean individual risk for goat *g* = 1 for different specificity and mean initial FEC.

The individual’s specificity seems to increase the Mean Individual Ingestion Risk (MIIR), as shown on Fig. 2. For a same initial mean FEC, one can note that the MIIR can make important jump, such as for specificity *a* = 0.0258 or *a* = 0.0505 for the first and second flock and for an initial mean FEC equals to 6*e*^−3^ or 4*e*^−3^. As will be discussed in the next section, there is a high variability in the individual’s ingestion risk, which can be extremely high in some cases. Except for these two cases, the mean individual’s risk distribution generally increases linearly with the specificity (*R*^2^ > 0.8).

### Individual’s risk distribution

**Figure 3:**
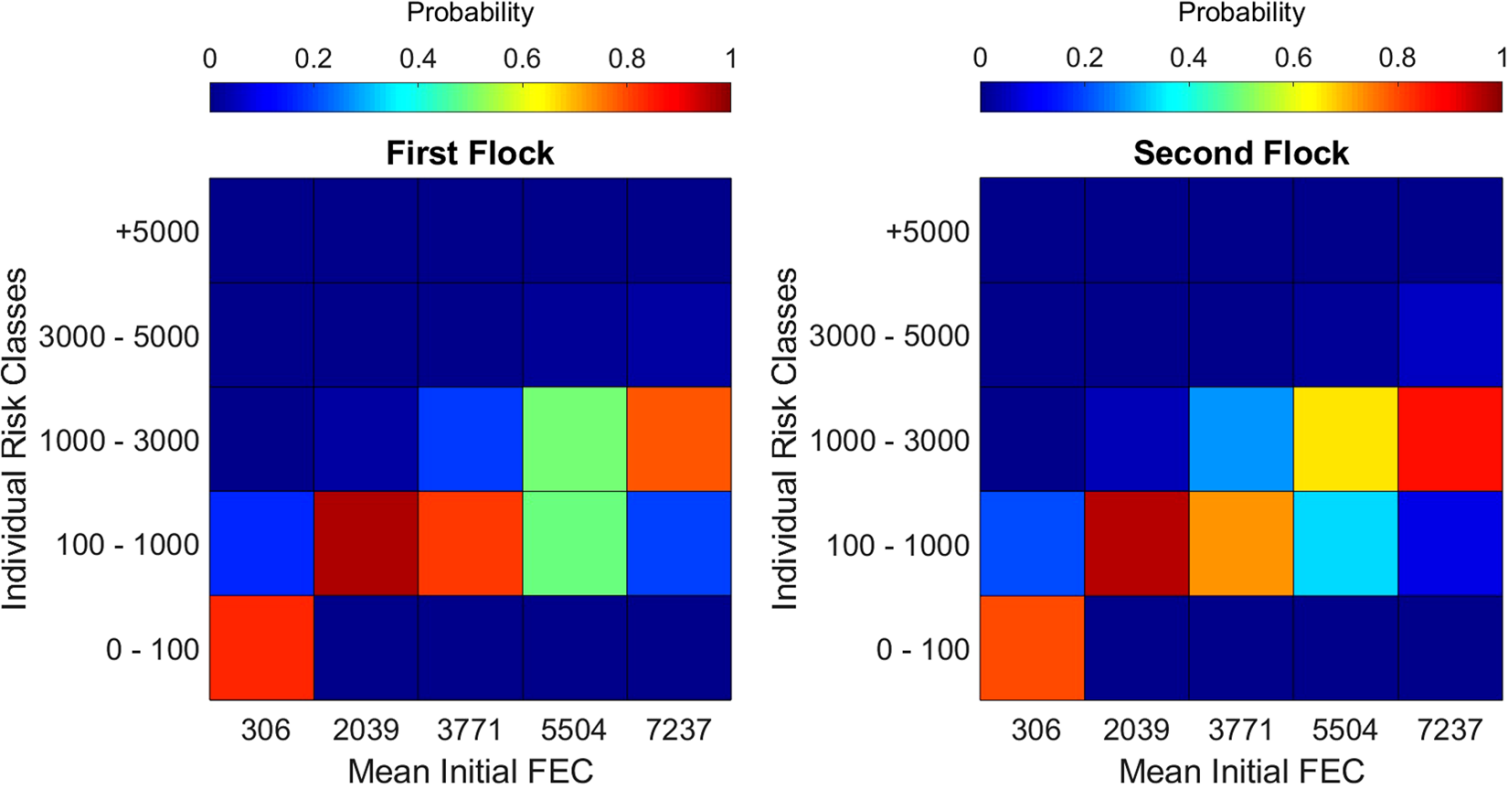
Individual’s risk class probabilities as a function of the mean initial FEC. We constructed the class’ bounds by interpreting the risk as the number of ingested L3 and the different classes as the health status of the animal.

Our sample includes the 5,000 simulated risk values of the first goat *g* = 1, for a given initial FEC distribution, no matter the specificity value. The individual’s risk distribution is right-skewed with a long tail. In other words, an individual is generally exposed to low risk (relatively to the flock’s initial FEC) but high exposure is still possible. Even after a logarithmic transformation, the individual’s risk distribution is still right-skewed and the normality hypothesis is generally rejected (Kolmogorov-Smirnov test, 5% significance level). We displayed the individual’s risk classes as a function of the initial FEC parameters on Fig. 3. We divided risk values into five classes with increasing risk value from no/poor risk to extremely high risk, and gives the probability of being in each class given the mean initial FEC. We constructed the class’ bounds by interpreting the risk as the number of ingested L3 and the different classes as the health status of the animal. The five classes correspond to: no effect, low effect, acceptable, not acceptable, unmanageable. One can see that the individual risk is in general lower than the flock’s mean initial FEC and that individual risk is higher for the second flock. Finally, the individual risk naturally increases with the parameters of the initial FEC distribution. Indeed, the simulated MIIR increases linearly with the mean initial FEC (*R*^2^ > 0.9). For the first (second) flock, the MIIR increases by 0.20 (0.24) with the initial mean FEC.

### Flock’s risk distributions

**Figure 4:**
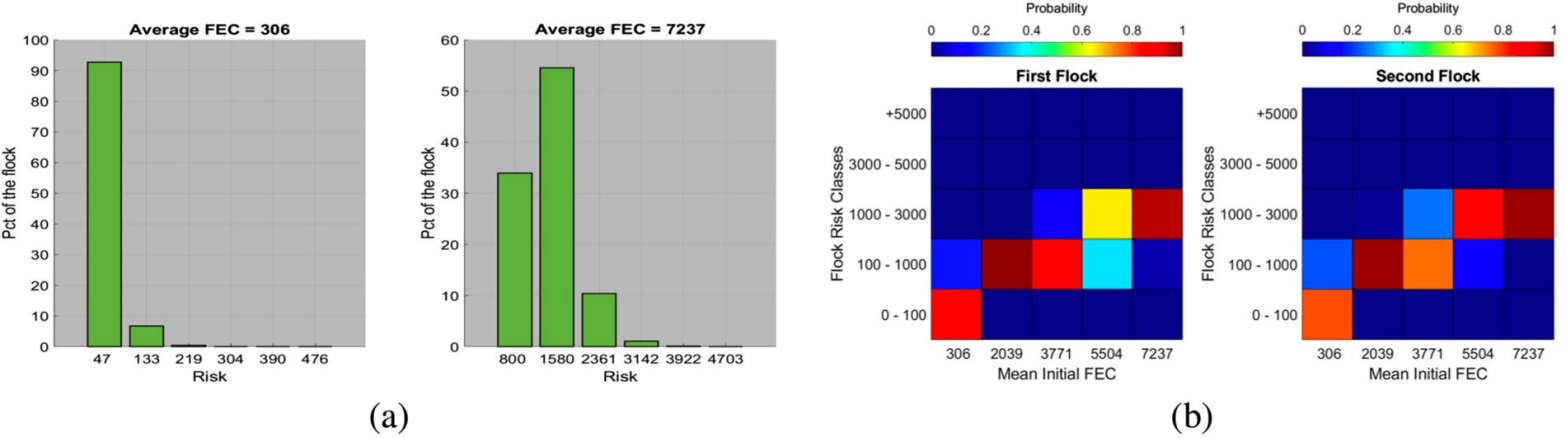
(**a**) Examples of the flock’s risk distribution for two different initial FEC distributions. On the left-hand, the average FEC of the flock during the simulation was 1942 and 34464 for the right-hand side. The x-axis gives the risk values and the y-axis gives the percentage of the flock in each risk class. To obtain these histograms, we used simulated values using log-normal distributions fitted with our data. (**b**) Mean flock’s risk class probabilities as a function of the initial FEC parameters. We constructed the class’ bounds by interpreting the risk as the number of ingested L3 and the different classes as the health status of the animal.

We now study the risk distribution at the flock scale. A sample becomes the simulated risk of the entire flock for a given initial FEC distribution, therefore, there are 250,00 samples. The flock’s risk distribution is again right-skewed but this time a log transformation is sufficient to ensure normality of the sample in more than 99.8% of the cases (Kolmogorov-Smirnov test, 5% significance level). See Fig. 4(a,b) for examples of flock’s risk distribution and mean flock’s risk class probabilities. Again, the average risk of the flock is generally lower than the mean initial FEC. The flock’s risk distribution also increases with the mean initial FEC (*R* ≥ 0.9). For the first (second) flock, the mean simulated flock’s risk increases by 0.20 (0.23) with the mean initial FEC.

### Feces’ spatial distribution

#### Dispersion

**Figure 5:**
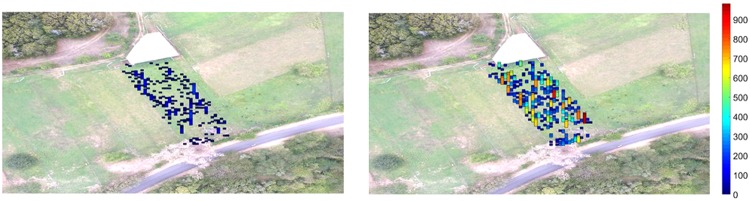
Examples of the feces’ spatial distribution for the second flock. On the left-hand, mean of the initial FEC distribution was 306 and 7236 on the right-hand. These examples are for the pasture of the second flock.

The feces’ spatial distribution is highly aggregated, resulting in most quadrats having a low number of L3 and a few quadrats having a high value (see Fig. 5 for examples).

Statistically, the hypothesis of an aggregated dispersion is confirmed using the index of dispersion^17^. Note that the hypothesis of an aggregated distribution is rejected one time out of 5000 for the first flock. This corresponds to a case where very few L3 were present on the pasture (only 83). The aggregation index generally increases with the mean of the initial FEC distribution but there is too much variation to fit a linear model.

#### Sampling L3 in the pasture

We used the simulated spatial distribution of L3 to study the efficacy of uniform random sampling to estimate the average number of L3 per m^2^ on a pasture. We first virtually divided the pasture into 25 zones of approximately 43 m^2^ and we then randomly selected *n* = 1, …, 5 sampling units (20 × 20 cm quadrats) per zone. The sampling units were chosen independently for each simulated spatial distribution of L3. Due to the aggregated nature of the feces’ spatial distribution, L3 are also highly aggregated on the pasture, which complicates sampling. Indeed, L3 are rarely found in the sample, which decreases the sample mean and in practice can lead us to mistakenly conclude that the pasture is not infected. The estimated average number of L3/m^2^ generally has a very poor quality, with more than 96% of error for each flock and all the tested number of samples. Note that increasing the number of samples has very little effect; for example, for the first flock, the average percentage of error is 96.7% with 25 samples and still 96.1% with 125 samples. The percentage of error is obtained as:

$$error=100\times \frac{|\tilde{x}-x|}{x}\mathrm{.}$$

Where *x* is the true number of L3/m^2^, $\tilde{x}$ is the estimated number of L3/m^2^ and |.| is the absolute value function.

**Figure 6:**
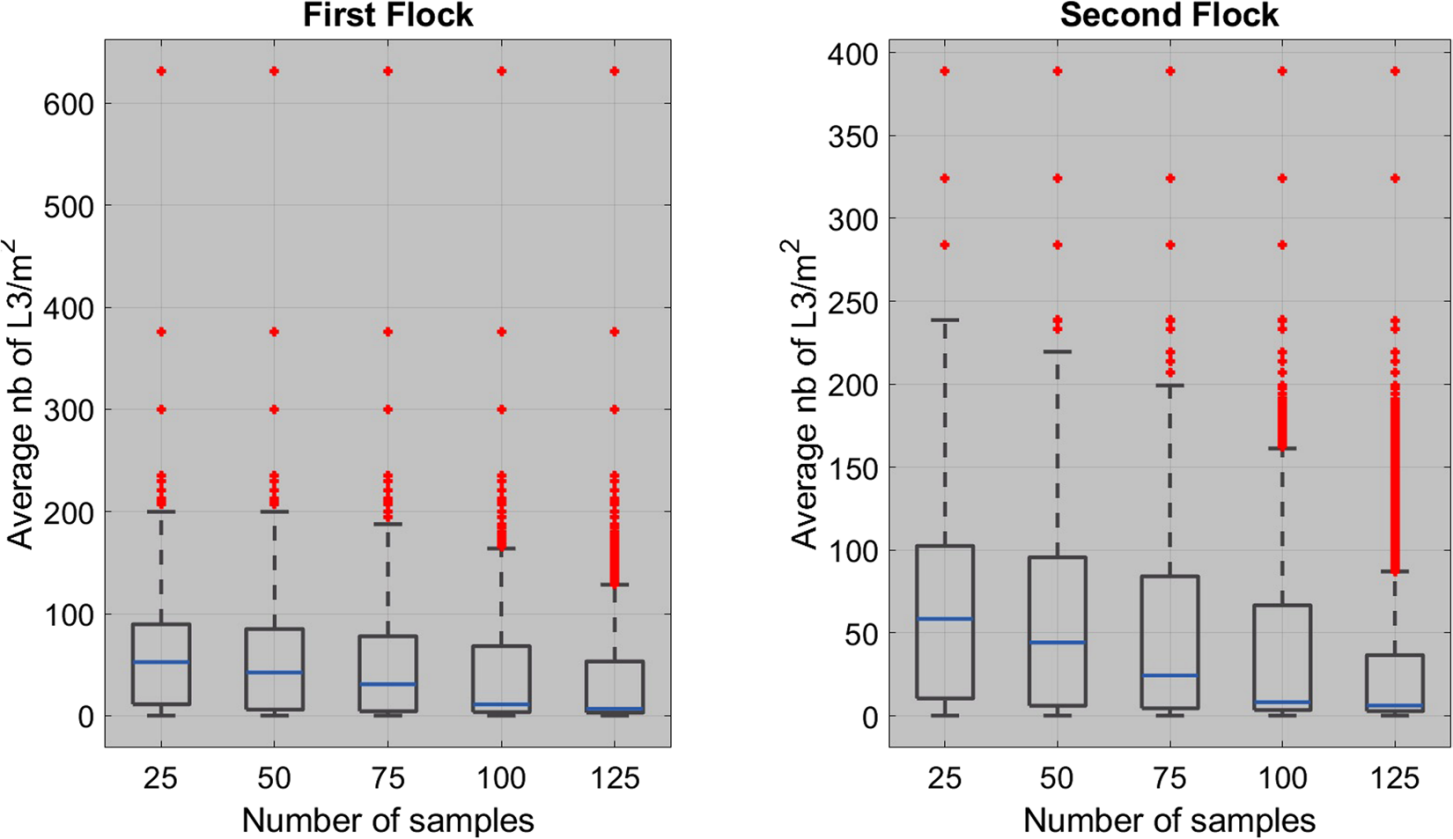
Distribution of the average number of L3 per m^2^ in the cases where no L3 were present in all of the sampled quadrats. There is one box-plot for each initial number of samples. The blue line gives the median value and the box indicate the 25th and 75th percentiles.

For both flocks, in more than 43%, 19%, 9%, 4% and 2% of the simulations, no L3 were present in the 25, 50, 75, 100 or 125 sampled quadrats. In other words, in more than 43% of the cases, the pasture was mistakenly classified as not infected when using 25 samples. As one can see in Fig. 6, the blank samples does not necessarily happen in cases where the density of L3/m^2^ was small.

### Number of goats

Finally, we varied the number of goats in the pasture from 10 to 30 using simulation scheme S2. For a fixed pasture size, the average risk of the flock naturally increases with the number of goats present on the pasture. We fitted a linear model with interactions, explaining the average risk of the flock as a function of the number of goats and of the mean initial FEC:

$$R\tilde{i}sk\simeq \alpha +{\beta }_{1}\ast nG+{\beta }_{2}\ast {I}_{F\tilde{E}C},\,+\gamma \ast nG\ast {I}_{F\tilde{E}C}$$

This model explains most of the variance with *R*^2^ = 0.997 for the first flock and *R*^2^ = 0.99 for the second flock. The estimated parameters are available in Table 1.

**Table 1:** Parameters of the linear model with interactions.

	Estimate	SE	tStat	pValue
**First Flock**				
*α*	5.9254	13.5067	0.4387	0.6618
*β*_1_	0.0224	0.0030	7.4720	0.0000
*β*_2_	−0.2862	0.6464	−0.4428	0.6589
*γ*	0.0099	0.0001	68.5451	0.0000
**Second Flock**				
*α*	5.9254	13.5067	0.4387	0.6618
*β*_1_	0.0224	0.0030	7.4720	0.0000
*β*_2_	−0.2862	0.6464	−0.4428	0.6589
*γ*	0.0099	0.0001	68.5451	0.0000

The linear model predicts the mean flock’s risk as a function of mean initial FEC and the number of goats on the pasture.

## Discussion

We studied the problem of L3 ingestion risk for goats managed under rotational grazing. As far as directly observing the number of ingested L3 is unmanageable, we proposed the first modeling approach to quantitatively characterise the ingestion risk distribution. This model is based on an estimation of the quadrat occurrence frequencies at the pasture scale and the hypothesis that the number of ingested L3 by an individual on a given position is proportional to the number of L3 on the ground and the time spent on this position:

$$Nb{I}_{g}={c}_{1}\ast \sum _{q=1}^{nQ}\,{t}_{gq}\ast NbL{3}_{q}={c}_{2}\ast Ris{k}_{g},$$

where *NbI*_*g*_ is the number of ingested L3 by goat *g* and *NbL*3_*q*_ is the number of L3 on quadrat *q*. However, this framework does not allow us to compute the constants *c*_1_ or *c*_2_ but we believe that characteristics of the risk distribution and of the number of ingested L3 might be similar. In other words:

1. The individual’s grazing behavior (here the specificity) influences the daily number of ingested L3.
2. Distribution of the number of daily ingested L3 at the individual and flock scale is right-skewed and is in any case non-homogeneous.
3. The average individual and flock daily number of ingested L3 increase with the average initial FEC and number of goats in a fixed pasture.
4. The spatial distribution of L3 on the ground is highly aggregated and inhomogeneous, which certainly explains the risk distribution and makes estimation difficult via *on-ground* sampling.

These conclusions echo the results from the literature that were described in introduction but our work brings quantitative informations that were missing for model design. More generally, our modeling framework shows that not only an animal’s body conditions and genetic but also mechanisms of infection (feces drop - survival - ingestion) and grazing spatial process are responsible for individual variation. We believe that better quantifying timing and number of ingested L3 can improve the modeling of the host parasite interaction and will eventually improve the host’s management. In addition, models that simulate infection dynamic should benefit from our framework to link worm burden reduction and short/long term infection risk. We ran our model in a particular situation (rotational grazing) and with a particular species (goats) and, therefore, the conclusions may change with other settings. In particular, it should be very interesting to compare the conclusions with sheep, who have a totally different behavior, or to compare with young animals who do not know the spatial distribution of food on the pasture.

To consolidate these conclusions, our modeling framework can be first combined with higher picture frequency^18^, which will certainly provide a better estimation of the quadrats occurrence frequencies. Our framework can also benefit from higher resolution images, allowing individual detection and activity detection (rest or ruminate - eat - walk), which in turn will allow better risk estimation. We showed that individual time per quadrat can be approximated using occurrence frequencies but other materials, such as GPS^19^, can allow us to compute the individual time per quadrat and an accelerometer will allow us to record activity^20^. Other parameters can also be accounted for, such as the pasture topology combined with the daily precipitation to account for L3 movement via water^21^.

Finally, this framework could also be used as a management tool. Indeed, if the time per quadrat can be recorded in real or near-real time and FEC are updated regularly, then one can follow the risk distribution per individual and concentrate management effort only on animals with higher risks. One can also identify areas in the pasture with the highest probability of having a large number of L3 and, for example, try to decrease the egg’s development with plant secondary metabolites^22^ or by mechanically removing the eggs or dry feces. This framework can also be used to better understand animal behavior and to identify areas that are more attractive for animals, and then enlarge these areas through agronomic improvement.

## Methods

### Aerial photos using drone

We recorded the positions of the animals in the two flocks, who were managed under rotational grazing. Each flock alternates grazing on five different pastures of approximately 1.2 ha each (10 pastures in total), spending 7 days per pasture, from Friday until Thursday of the next week, before moving to the next adjacent pasture. For example, when a flock is on pasture P1 on a given day, it will be again on pasture P1 five weeks later. The first flock (F1) was composed of 21 goats and 27 kids, while the second flock (F2) was composed of 22 goats and 28 kids. Pastures are located (16°20 N; 61°20 W) at the INRA-PTEA farm under a tropical climate. Each pasture is connected to a free access shelter where the animal can spend the night and have access to water. Every morning at around 8 am, a concentrate supply was distributed in the shelter.

We recorded the positions of the goats on the two pastures during four consecutive days, from April 10^th^ 2017 to April 13^th^ 2017. We replicated this study one month later, thus when each flock was back on the initial pasture, from May 15^th^ 2017 to May 18^th^ 2017. Due to losses in flock F2, each flock was composed of 27 kids during the second observation week while the number of adult goats remained constant.

We flew a drone (Phatom 3 of the Dji brand) every 15 to 30 minutes and take a picture of the two flocks, from 9 am to 6 pm at about 50 m from the ground. No photo was taken during a one-hour break around noon. In total, 122 (mean = 30.5/day, std = 9.2) and 120 (mean = 30/day, std = 9.5) photos were taken during the first week for the first and second flock; and 113 (mean = 28.25/day, std = 5.7), 115 (mean = 28.75/day, std = 5.3) during the second week. The photos did not provide direct geographical information, so we used the QGis software v 2.18.5 to point on each of the goats (not the kids) present in the pasture and then extract their spatial coordinates. Note that we did not differentiate the activity of the animals (feeding, sleeping, walking) because this was too difficult based on a picture taken from behind. All of the statistical analyses were performed using Matlab.

We virtually delimited several zones in the pastures, depending on the predominant grass species. We delimited the pasture of the first flock, denoted P1, into ten different zones and the pasture of the second flock, denoted P2, into seven different zones. The dominant grass species on each of the zone is provided in Supplementary Table S1 and a visual of the zones is provided in Supplementary Fig. S3. The species present in the two pastures are *Panicum maximum*, *Digitaria Decumbens*, *Dichanthium sp*., *Paspalum dilatatu*, *Kyllinga sp*., *Brachiaria mutica purpuresens*, *Achyranthes aspera*, *Sida acuta*, *Digitaria swazilandensis*, and *Sporobolus indicus*. Note that the zones are highly heterogeneous and several species, if not all, can be found inside a same zone. Meteorological data were collected from the nearest weather station 1 km away, from the on-line platform https://intranet.inra.fr/climatik_v2/ClimatikGwt.htmlClimatik (accessed on November 29^th^, 2017. Meteorological station 97118002).

### A simulation framework of goats and L3 spatial distribution

We proposed a simulation framework because it allows us to describe the time that each animal spends on the different locations of the pasture during the first grazing week. From this, we simulated the number of eggs that were deposited on each of these locations and the effect of environmental factor on the development of eggs into infective L3. Finally, based again on the locations and time spend when the flock re-enter the pasture, we derived an infection risk for each animal. Schematic representations of our simulation frameworks are proposed in Supplementary Figs S4 and S5.

#### First week on the pasture

Notations: We virtually divided each pasture into a regular grid of square quadrats of 1 m each. The number of quadrats in a pasture is denoted *nQ* and the number of goats on the pasture *nG*. Let us define *t*_*gq*_, the time (not otherwise specified, all times are in minutes) spent by goat number *g* ∈ {1, …, *nG*} on quadrat number *q* ∈ {1, …, *nQ*}. In addition, *T*_*g*_ is the total time spend on the pasture by individual number *g*, while *C*_*q*_ is the accumulated time spent on quadrat number *q*, such that we have:

$${T}_{g}=\sum _{q=1}^{nQ}\,{t}_{gq},\,{C}_{q}=\sum _{g=1}^{nG}\,{t}_{gq}\,{\rm{and}}\,\sum _{g=1}^{nG}\,{T}_{g}=\sum _{q=1}^{nQ}\,{C}_{q}.$$

We will use ${t}_{gq}^{1}$, ${T}_{g}^{1}$, ${C}_{q}^{1}$ for the first week and ${t}_{gq}^{2}$, ${T}_{g}^{2}$, ${C}_{q}^{2}$ for the second week on the pasture. For example, ${T}_{g}^{1}$ is the total time spent on the pasture during week 1 by goat number *g* and ${C}_{q}^{2}$ is the cumulative time spent on quadrat *q* during week 2.

Fecal deposit: We hypothesis that feces are deposited in clumps and that the probability of one fecal clump being deposited on quadrat *q* is equal to $\frac{{t}_{gq}}{{\sum }_{q^{\prime} =1}^{nQ}\,{t}_{gq^{\prime} }}$, for individual *g*. Thereby, individuals are more likely to drop feces on the quadrats that they visited the most.

We used the work from Takeuchi *et al*.^23^ to estimate the number of fecal clumps per animal over a period of 11 hours, which follows a normal distribution with mean 8.5 and standard deviation 3.6. We used the work from Ortega-Jimenez *et al*.^24^ to estimate the weights of feces per individual over a period of 5 days (dry matter), which follows a normal distribution with mean 1900.2 grammes and standard deviation 688.11 grammes. We scaled these probability distributions to simulate a weight of feces and number of clumps per animal given the time spend on the pasture *T*_*g*_. We hypothesis an equal weight distribution among clumps. Finally, we initialised the number of eggs per gram of feces (Fecal Eggs Count, FEC) for each animal using the work from Mahieu *et al*.^25^, where they sampled 15 farms in Guadeloupe. We fitted a negative binomial distribution to each of the 15 farms to derive a realistic numerical domain for the *r* and *p* parameters of the negative binomial distribution. The domain for *r* is [0.19756; 1.4644] and [3.33*e*^−5^; 8.2199*e*^−4^] for *p*.

Note that we divided each quadrat into smaller 20 cm square quadrats and recorded the locations of each clump to be used in other section.

Life cycle of the free living stages: We used the work from Rose *et al*.^26^ to simulate the effects of environmental factors (temperature, total daily precipitation and total daily evapotranspiration) on the development of eggs into infective L3. When the flock re-enters the pasture one week later, on average, only 0.1% of the eggs developed into infective L3 and survived at this time.

#### Second week on the pasture (i.e. five weeks later)

Ingestion risk: Let define *E*_*z*_ as the time needed for one goat to consume all the resources of a quadrat located zone *z*. We supposed that for each minute on a quadrat, a proportion $\frac{1}{{E}_{z}}$ of the available L3 are ingested by the goat. For each of the ${C}_{q}^{2}$ minutes on a given quadrat *q*, a goat *g* is selected with probability ${t}_{gq}^{2}{C}_{q}^{2}$ and received $\frac{1}{{E}_{z}}$ of the total number of L3 still present on the quadrat. This process is repeated for each quadrat and the number of received L3 per goat is updated. The goat selection process is arbitrary, so we repeated the process independently 500 times and used the average number of received L3 per goat.

It is hazardous to interpret this quantity directly as the number of ingested L3 and we preferred to define it as a risk, which can nonetheless be a first approximation of the number of ingested L3. In our opinion, the number of ingested L3 is likely to be function of this risk. Note that risk can either be considered as a continuous variable or discrete variable when rounded.

### Fitting the model to the data

#### Individual grazing time

The drone only provides the positions of individuals on a discrete time step, thus the *t*_*gq*_ are not directly observable and need to be estimated. We first estimated the cumulative flock grazing time for week 1 and 2 by taking the integral of the extrapolated number of goats as a function of time. This represents the sum of the grazing time of each goat over the 4 days of the experiment. We finally divide this cumulative grazing time by the number of goats to get the average grazing time per individual, denoted *mg*^*w*^, for week number *w* = 1, 2. Finally, to consider variation among individuals during the simulation, the grazing times *T*_*g*_ were drawn from a normal distribution of mean *mg*^*w*^ and variance *σ* * *mg*^*w*^ at the beginning of each run.

#### Occurrence frequency

We computed the frequency at which a goat was observed on a quadrat; i.e., the number of time a goat was detected on the quadrat divided by the number of pictures took from the drone. Let *f*_*q*_ denote the normalised frequency for quadrat *q*, such that ${\sum }_{q=1}^{nQ}\,{f}_{q}=1$.

#### Time spent on a quadrat

Let define {*λ*_*g*1_, …, *λ*_*gnQ*_} a set of normalised weights such that for all goat *g*:

$$\sum _{q=1}^{nQ}\,{\lambda }_{gq}=1\,{\rm{and}}\,{\rm{for}}\,{\rm{all}}\,{\rm{quadrat}}\,q,\,{t}_{gq}={\lambda }_{gq}{T}_{g}.$$

*λ*_*gq*_ is simply the proportion of time goat *g* spends on quadrat *q*. There are unfortunately no ways to estimate this weight using our dataset or any published data and, therefore, the weights will be drawn randomly. These weights depict some aspect of the animals’ behavior. For example, when they are all equal (i.e., *λ*_*g*1_ = … = *λ*_*gnQ*_), the goat tends to explore all the quadrats. In contrast, when only a few of the weights are non-zeros, the goat is very specific and only spends time on a few quadrats. To simulate these different behaviors, we proposed to use a geometric distribution of parameter *a* ∈ [0.001; 0.1]. When *a* = 0.001, the time distribution is uniform and all weights are almost equal. When *a* = 0.1, the time distribution is highly heterogeneous, with the most visited quadrat monopolising 10% of the cumulative grazing time; and nearly 96% of the quadrats are visited for less than 1% of the cumulative grazing time (i.e., *t*_*gq*_ ≤ 0.01 * *T*_*g*_ for 96% of the quadrats). Once the weights are fixed, we simply used *t*_*gq*_ = *λ*_*gq*_ * *T*_*g*_. *a* will be called the specificity parameter.

#### Simulation scheme S1

We used this framework to simulate the individual time per quadrat during week 1 and 2, location of feces clumps and development from eggs to L3 to finally estimate the ingestion risk for each individual. To run the simulations, we defined domains of five equally spaced values for the initial FEC parameters (*r*, *p*) and specificity parameter *a*. We used a constant value of *p*, equals to the mean value estimated on the dataset from^25^. We selected five values of *r* such that the corresponding means of the initial FEC distributions cover the domain of the observed mean in the dataset. We used *p* = 32.55*e*^−5^, *r* = (0.099646, 0.66386, 1.2281, 1.7923, 2.3565) and *a* = (0.0010.02575; 0.0505; 0.07525; 0.1). For each of the parameter combination, we ran 1000 simulations. There is thus 5 * 5 = 25 parameter combinations and 125*1000 simulations were run in total. A simulated trajectory thus includes:

- An individual initial FEC value, drawn from a negative binomial with parameter (*r*, *p*) and mean $\frac{r\ast \mathrm{(1}-p)}{p}$.
- An individual specificity value *a*_*g*_, for *g* = 1, …, *nG*.
- An individual time per quadrats for the first grazing week, ${t}_{gq}^{1}$ for *g* = 1, …, *nG* and *q* = 1, …, *nQ*.
- A number of eggs per quadrat after one grazing week.
- A number of surviving L3 per quadrat at the beginning of the second grazing week.
- An individual time per quadrat for the second grazing week, ${t}_{gq}^{2}$ for *g* = 1, …, *nG* and *q* = 1, …, *nQ*.
- An individual ingestion risk value.

**Figure 7:**
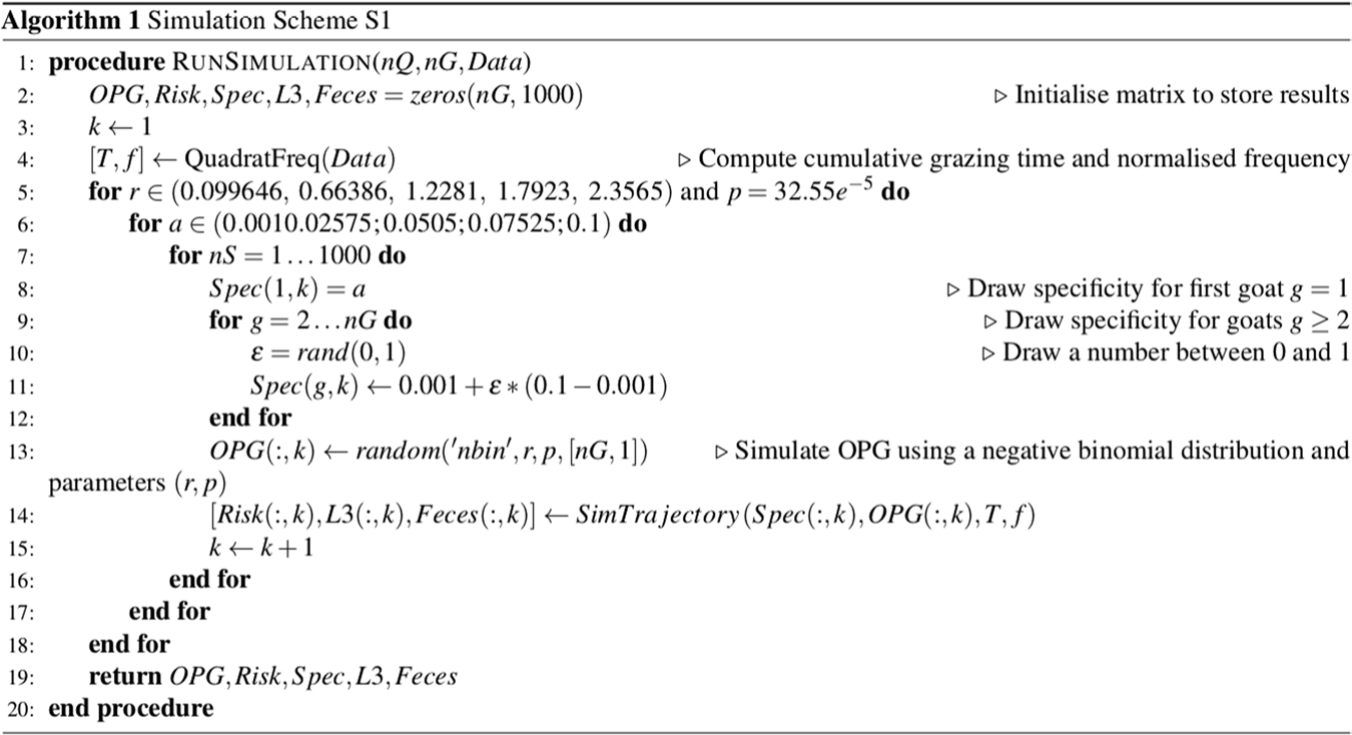
Simulation scheme S1 in pseudo code.

Note that for each run, parameter *a* was prefixed for the first goat and drawn randomly for the others. For example, for the 1000 first simulations, we used (*r*, *p*) = (0.099646, 32.55*e*^−5^), we used *a* = 0.001 for the first goat *g* = 1 and drawn *a* randomly for the other goats *g* > 1. Simulated risk of the first goat will be used to analyze the risk distribution at the individual scale and the effect of the specificity parameter. See Fig. 7 for a pseudo code.

#### Simulation scheme S2

We used a simplified scheme for the other experiments by choosing specificity parameter randomly for all goats *g* ≥ 1. In this case, there are 5*1000 simulations.

We used Matlab to run the simulation and all of the codes are available in the supporting information All mathematical notations are summarised in Supplementary Table S2.

### Ethical approval and informed consent

The animals were already present at the farm and because the drone flew at an altitude of 50 m, the animals’ behavior and welfare were not impacted. Our experiment did not require any intervention of any type on the animals. The animals were manage under normal condition at the PTEA farm, respecting French and European laws.

## Electronic supplementary material

Supplementary tables and figures

## Data Availability

The datasets generated during the current study and Matlab codes are available in the Larval_Intake https://github.com/mbonneau13/Larval_Intake.git.

## References

[1] Nieuwhof G, Bishop S (2005). Costs of the major endemic diseases of sheep in great britain and the potential benefits of reduction in disease impact. Animal Sci..

[2] Aumont G, Gruner L (1989). Population evolution of the free-living stage of goat gastrointestinal nematodes on herbage under tropical conditions in guadeloupe (french west indies). Int. journal for parasitology.

[3] O’Connor LJ, Walkden-Brown SW, Kahn LP (2006). Ecology of the free-living stages of major trichostrongylid parasites of sheep. Vet. parasitology.

[4] van Dijk J, de Louw M, Kalis L, Morgan E (2009). Ultraviolet light increases mortality of nematode larvae and can explain patterns of larval availability at pasture. Int. J. for Parasitol..

[5] Bambou J-C (2009). Peripheral immune response in resistant and susceptible creole kids experimentally infected with haemonchus contortus. Small Rumin. Res..

[6] Houdijk J (2008). Influence of periparturient nutritional demand on resistance to parasites in livestock. Parasite immunology.

[7] Cornell SJ, Isham VS, Grenfell BT (2004). Stochastic and spatial dynamics of nematode parasites in farmed ruminants. Proc. Royal Soc. London, Ser. B: Biol. Sci..

[8] Tallis G, Leyton M (1969). Stochastic models of populations of helminthic parasites in the definitive host. i. Math. Biosci..

[9] Cornell S, Isham V, Smith G, Grenfell B (2003). Spatial parasite transmission, drug resistance, and the spread of rare genes. Proc. Natl. Acad. Sci..

[10] Gaba S, Cabaret J, Ginot V, Silvestre A (2006). The early drug selection of nematodes to anthelmintics: stochastic transmission and population in refuge. Parasitol..

[11] Molento MB, Buzatti A, Sprenger LK (2016). Pasture larval count as a supporting method for parasite epidemiology, population dynamic and control in ruminants. Livest. Sci..

[12] Baumont R, Prache S, Meuret M, Morand-Fehr P (2000). How forage characteristics influence behaviour and intake in small ruminants: a review. Livest. Prod. Sci..

[13] Newman J. A., Parsons A. J., Thornley J. H. M., Penning P. D., Krebs J. R. (1995). Optimal Diet Selection by a Generalist Grazing Herbivore. Functional Ecology.

[14] Marion G, Swain DL, Hutchings MR (2005). Understanding foraging behaviour in spatially heterogeneous environments. J. Theor. Biol..

[15] Smith L, Marion G, Swain DL, White P, Hutchings MR (2009). Inter-and intra-specific exposure to parasites and pathogens via the faecal–oral route: a consequence of behaviour in a patchy environment. Epidemiol. infection.

[16] Fox NJ, Marion G, Davidson RS, White PC, Hutchings MR (2013). Modelling parasite transmission in a grazing system: the importance of host behaviour and immunity. PloS one.

[17] Kathirgamatamby N (1953). Note on the poisson index of dispersion. Biom..

[18] Benvenutti M (2015). The use of image analysis to determine the number and position of cattle at a water point. Comput. Electron. Agric..

[19] Buerkert A, Schlecht E (2009). Performance of three gps collars to monitor goats’ grazing itineraries on mountain pastures. Comput. Electron. Agric..

[20] Barwick J, Lamb DW, Dobos R, Welch M, Trotter M (2018). Categorising sheep activity using a tri-axial accelerometer. Comput. Electron. Agric..

[21] Stromberg BE (1997). Environmental factors influencing transmission. Vet. Parasitol..

[22] Marie-Magdeleine C, Mahieu M, Philibert L, Despois P, Archiméde H (2010). Effect of cassava (manihot esculenta) foliage on nutrition, parasite infection and growth of lambs. Small Rumin. Res..

[23] Takeuchi Y, Kikusui T, Mori Y (1995). Changes in the behavioral parameters following the lipopolysaccharide administration in goats. J. Vet. Med. Sci..

[24] Ortega-Jimenez E (2005). Intake and milk production of suckling creole goats reared at pasture in humid tropics according to the post-grazing residue management. Small Rumin. Res..

[25] Mahieu M, Ferré B, Madassamy M, Mandonnet N (2014). Fifteen years later, anthelmintic resistances have dramatically spread over goat farms in guadeloupe. Vet. Parasitol..

[26] Rose H, Wang T, van Dijk J, Morgan ER (2015). Gloworm-fl: A simulation model of the effects of climate and climate change on the free-living stages of gastro-intestinal nematode parasites of ruminants. Ecol. Model..

