# Supplementary material for: Goats worm burden variability also results from non-homogeneous larval intake
Source: Sci Rep. 2018 Oct 30;8:15987. doi: 10.1038/s41598-018-34338-2 (PMC6207733; doi:10.1038/s41598-018-34338-2)
Supplement: Supplementary file 1 — Supplementary tables and figures [file 41598_2018_34338_MOESM1_ESM.pdf]

Goats worm burden variability also results from non-homogeneous larval intake.

Mathieu Bonneau[1,*], Jean-Christophe Bambou[1], Nathalie Mandonnet[1], Rémy Arquet[2], and Maurice Mahieu[1]

[1]INRA - URZ, Animal Genetic, Petit-Bourg, 97170, Guadeloupe - French West Indies

[2]INRA - UE PTEA, Petit-Bourg, 97170, Guadeloupe - French West Indies

[*]Corresponding author: mathieu.bonneau@inra.fr

Supplementary Tables

Zone Number	Weed Species
**Pasture Flock 1**	
Z0	Digitaria Decumbens, Brachiaria mutica purpuresens, Dichanthium sp.
Z1	Paspalum dilatatu
Z2	Kyllinga sp., Digitaria Decumbens
Z3	Achyranthes aspera
Z4	Dichanthium sp., Paspalum dilatatu
Z5	Sporobolus indicus
Z6	Dichanthium sp., Paspalum dilatatu
Z7	Achyranthes aspera
Z8	Mixture
Z9	Panicum maximum
**Pasture Flock 2**	
Z1	Panicum maximum
Z2	Brachiaria mutica purpuresens
Z3	Digitaria swazilandensis
Z4	Digitaria Decumbens
Z5	Achyranthes aspera
Z6	Dichanthium sp.
Z7	Dichanthium sp., Paspalum dilatatu

Table S1: Dominant weed species in each pasture.

Variable	Definition
$a \in [0.01; 0.1]$	Specificity: goat exploration behavior.
$g = 1, \ldots, nG$	Goat number.
nG	Number of goats in the flock.
$q = 1, \ldots, nQ$	Quadrat number.
nQ	Number of quadrats.
T_g	Total time on the pasture for goat g.
C_q	Cumulative time spend on quadrat q.
t_{gq}	Total time spends by goat g on quadrat q.
(r, p)	Parameters of the initial FEC distribution.
	We used a negative binomial distribution, with mean $\frac{r*(1-p)}{p}$.

Table S2: Principal mathematical notations.

Supplementary Figures

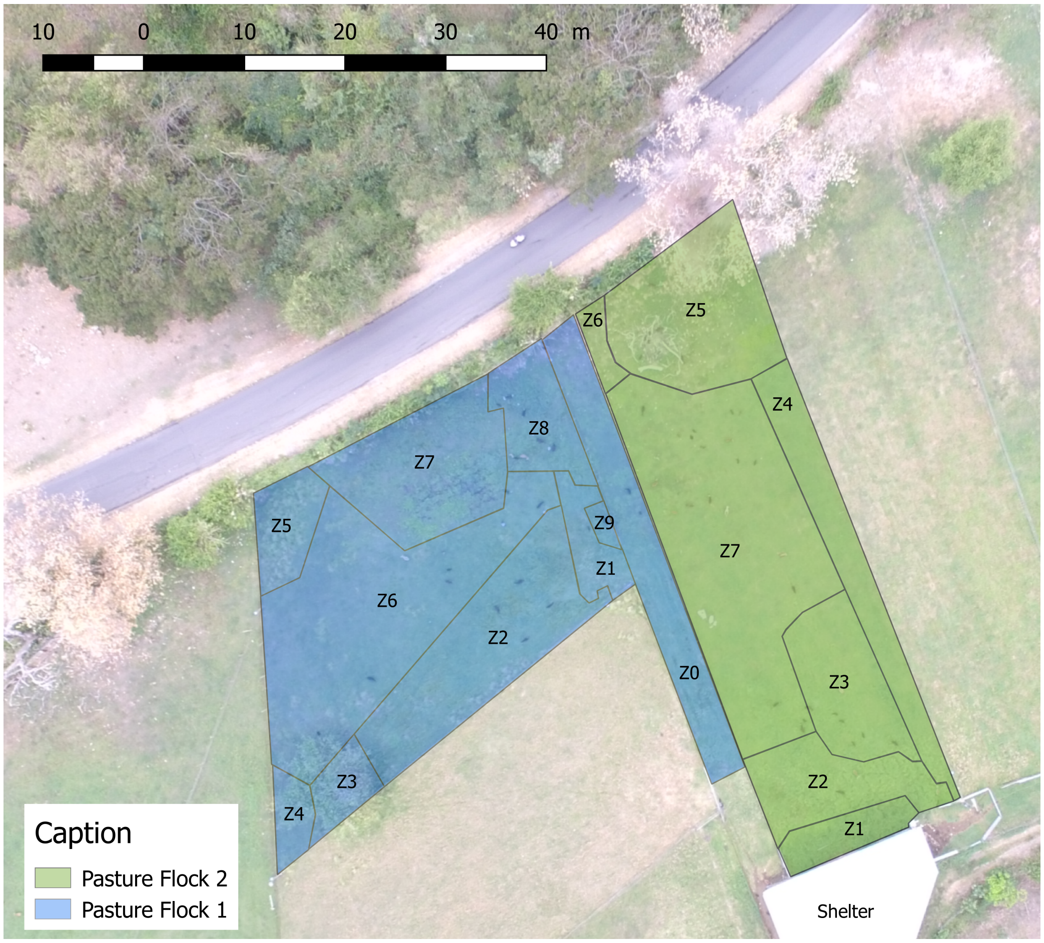

Figure S3: Location of the different zones. Zones are defined as part of the pasture with a particular dominant weed species.

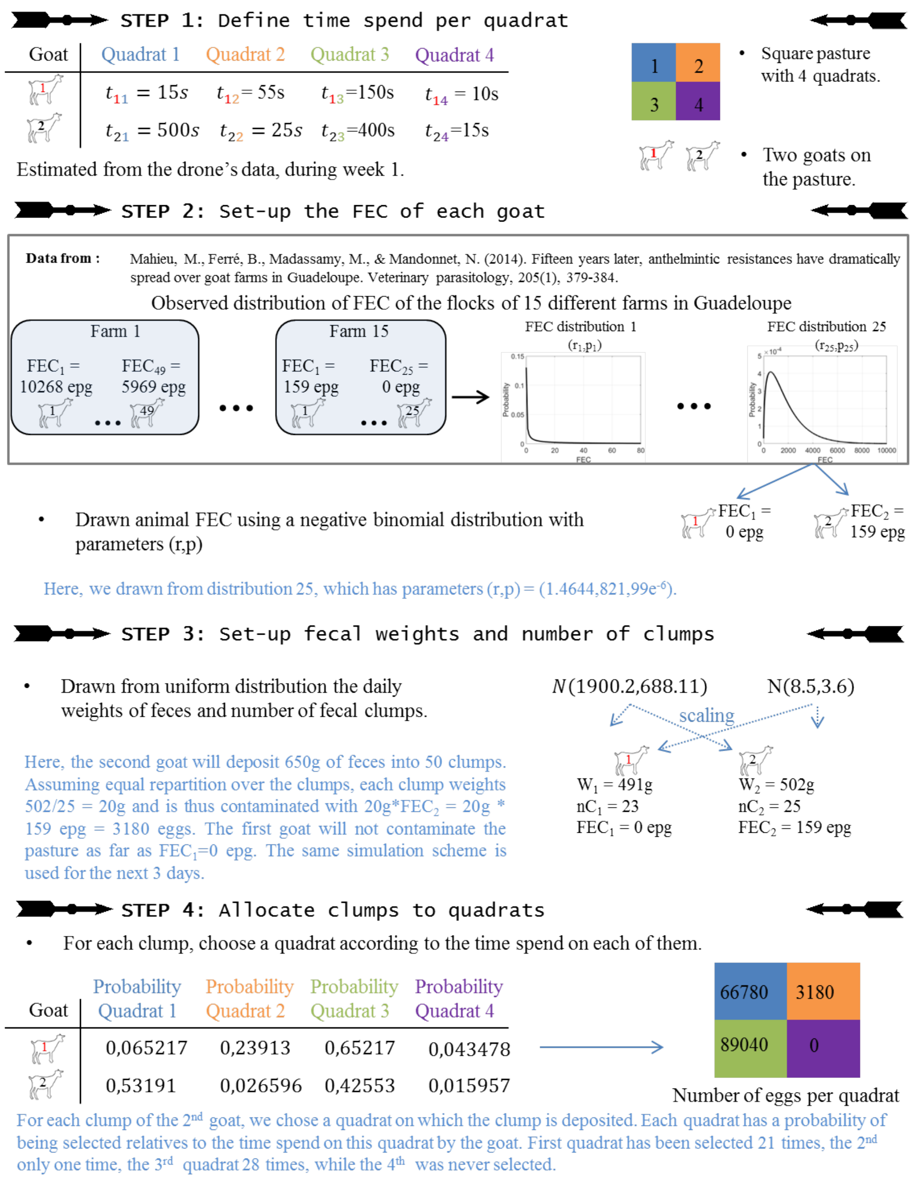

Figure S4: Schematic representation of the simulation scheme that was used to simulate the spatial distribution of eggs on the pasture after the first grazing week. Numerical values are given for illustration purpose only.

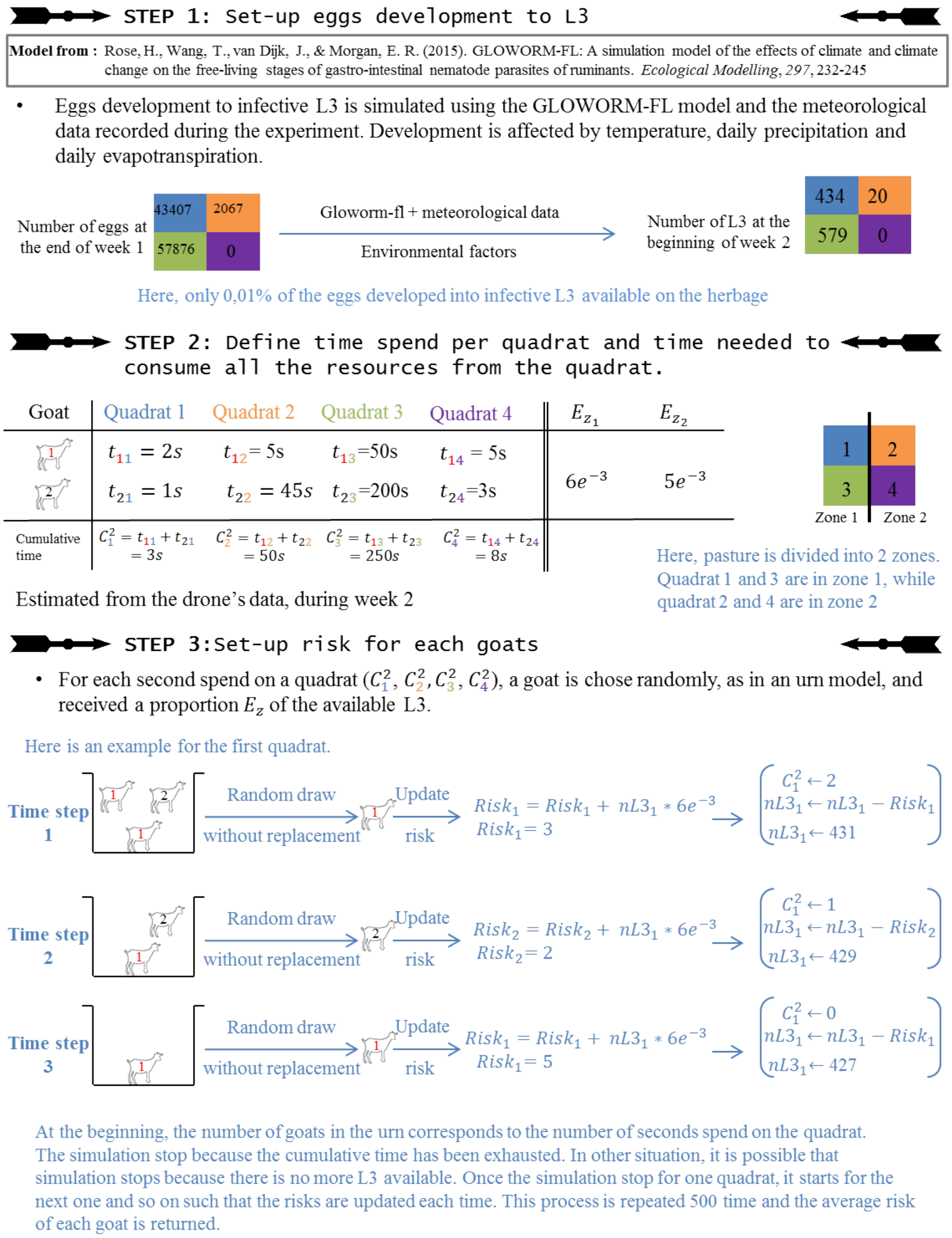

Figure S5: Schematic representation of the simulation scheme that was used to simulate the infection risk at the end of the second grazing week. Numerical values are given for illustration purpose only.
